# Gap-Free Tuning of Second and Third Harmonic Generation in Mechanochemically Synthesized Nanocrystalline LiNb_1−*x*_Ta_*x*_O_3_ (0 ≤ *x* ≤ 1) Studied with Nonlinear Diffuse Femtosecond-Pulse Reflectometry

**DOI:** 10.3390/nano14030317

**Published:** 2024-02-04

**Authors:** Jan Klenen, Felix Sauerwein, Laura Vittadello, Karsten Kömpe, Vasyl Hreb, Volodymyr Sydorchuk, Uliana Yakhnevych, Dmytro Sugak, Leonid Vasylechko, Mirco Imlau

**Affiliations:** 1 Department of Mathematics/Informatics/Physics, Osnabrueck University, 49076 Osnabrueck, Germany; 2 Research Center for Cellular Nanoanalytics, Osnabrueck (CellNanOs), Osnabrueck University, 49076 Osnabrueck, Germany; 3 Department of Biology/Chemistry, Osnabrueck University, 49076 Osnabrueck, Germany; 4 Department of Semiconductor Electronics, Lviv Polytechnic National University, 79013 Lviv, Ukraineleonid.o.vasylechko@lpnu.ua (L.V.); 5 Institute for Sorption and Problems of Endoecology, National Academy of Sciences of Ukraine, 13 Gen. Naumov St., 03164 Kyiv, Ukraine; 6 Scientific Research Company ‘Electron-Carat’, 79031 Lviv, Ukraine

**Keywords:** LNT, lithium niobate tantalate, harmonic nanoparticles, nonlinear optics, frequency conversion, harmonic generation, solid solution

## Abstract

The tuning of second (SHG) and third (THG) harmonic emission is studied in the model system LiNb${\;\;}_{1-x}$Ta${\;\;}_{x}$O${\;\;}_{3}$ (0≤x≤1, LNT) between the established edge compositions lithium niobate (LiNbO${\;\;}_{3}$, x=0, LN) and lithium tantalate (LiTaO${\;\;}_{3}$, x=1, LT). Thus, the existence of optical nonlinearities of the second and third order is demonstrated in the ferroelectric solid solution system, and the question about the suitability of LNT in the field of nonlinear and quantum optics, in particular as a promising nonlinear optical material for frequency conversion with tunable composition, is addressed. For this purpose, harmonic generation is studied in nanosized crystallites of mechanochemically synthesized LNT using nonlinear diffuse reflectometry with wavelength-tunable fundamental femtosecond laser pulses from 1200 nm to 2000 nm. As a result, a gap-free harmonic emission is validated that accords with the theoretically expected energy relations, dependencies on intensity and wavelength, as well as spectral bandwidths for harmonic generation. The SHG/THG harmonic ratio ≫1 is characteristic of the ferroelectric bulk nature of the LNT nanocrystallites. We can conclude that LNT is particularly attractive for applications in nonlinear optics that benefit from the possibility of the composition-dependent control of mechanical, electrical, and/or optical properties.

## 1. Introduction

The second- and third-order nonlinearities $\chi^{\left(2\right)},\chi^{\left(3\right)}$ are at the origin of a variety of important photophysical phenomena in nonlinear photonics, such as second and third harmonic generation [1]. Frequency converters based on $\chi^{\left(2\right)}$ and/or $\chi^{\left(3\right)}$ nonlinearities are widely used in quantum technologies of the first generation (e.g., tunable laser systems, all-optical signal processing) [2,3] and second generation (e.g., entangled photon pair sources [4,5]). Today, lithium niobate (LiNbO${\;\;}_{3}$, LN) and lithium tantalate (LiTaO${\;\;}_{3}$, LT) are important $\chi^{\left(2\right)}$ and/or $\chi^{\left(3\right)}$ materials due to their pronounced nonlinear optical coefficients, mechanical stability, and optical transparency in the near-ultraviolet to near-infrared spectral range [6,7]. The ferroelectric nature of LN and LT allows for periodic poling of the single crystals (e.g., [8,9]), facilitating efficient frequency conversion via quasi-phase matching in periodically poled lithium niobate (PPLN) and/or periodically poled lithium tantalate (PPLT). LN and LT have different melting points/stabilities at room temperature and different coercive field strengths, respectively, and thus offer different advantages: large mechanical and temperature (ferroelectric) stability (LN) versus electric field poling at low field strengths (LT).

Against this background, the use of $\chi^{\left(2\right)}$ and/or $\chi^{\left(3\right)}$ nonlinearities in the mixed ferroelectric system lithium niobate tantalate (LiNb${\;\;}_{1-x}$Ta${\;\;}_{x}$O${\;\;}_{3}$ LNT, space group R3c, LNT) appears to be interesting, particularly if one wants to combine the advantages of both edge compositions in the same system but also tailor nonlinear properties to the requirements of an individual application. For $\chi^{\left(2,3\right)}$-frequency conversion, LNT is of particular interest because of the zero crossing of the birefringence Δn at x≈0.94 at room temperature, which—so far—can be reached only in LT using temperature tuning [10,11]. The possibility of shifting the temperature dependence of the birefringence via its composition makes LNT an ideal material in setups for accurate temperature sensing as well [12,13].

The first attempt to access the nonlinear optical properties of LNT was reported by Sidorov et al., who showed the second harmonic intensity as a function of the composition of LNT ceramics with a microcrystalline structure (grain sizes ≈ 4–5 μm) and exposure to ns-laser pulses at a wavelength of 1064 nm [14]. Glass-embedded LNT crystallites with sizes of less than 50 nm were inspected in the work of Jaschin et al. [15]. Intensity and temperature dependence were validated in the hybrid LNT–glass system [15] and in LNT powders [16], respectively, with second harmonic generation as an example using the same pump conditions (1064 nm of a YAG:Nd pulse laser) and were found to be in accordance with the theory of nonlinear optics. Xue et al. calculated the composition dependence of the second-order nonlinear optical response of LNT single crystals with x in the vicinity of the edge compositions (0≤x≤0.05 and 0.8≤x≤1) and in comparison to LN and LT [17]. As a result, a remarkable decrease in the tensor elements of the second-order susceptibility is reported for increasing tantalum content at the fundamental wavelength of 1064 nm. According to the state of the literature, the presence of a $\chi^{\left(2\right)}$ nonlinearity can be effectively postulated for LNT, but information about the dispersion features as well as the generation of third harmonics is missing completely in the literature so far. Without this knowledge, the question about the suitability of LNT in the fields of nonlinear and quantum optics, in particular as a promising NLO material for frequency conversion, cannot be answered.

In this paper, the NLO properties of mechanochemically synthesized LNT nanocrystallites [18] are studied on the basis of SHG and THG using fundamental fs-pulses in the near-infrared spectral range from 1200 nm to 2000 nm. By using nanomaterials with a diameter 80≤d≤200 nm, i.e., smaller than the SHG and THG coherence length, that is, $d\ll l_{\mathrm{coh}}$, it is possible to study NLO properties without a phase-matching condition using the method of nonlinear diffuse fs-pulse reflectometry [19]. By using femtosecond laser pulse systems, in contrast with widely established pulse characterization based on Q-switched lasers (see, e.g., [20]), it becomes possible to adjust the energy per pulse, pulse duration, and pulse repetition rates to pulse peak intensities enabling the generation of higher harmonics. Furthermore, the time-averaged laser power, and thus the laser-induced thermal power, can be effectively adjusted to minimize thermal assisted effects, such as agglomeration, recrystallization/annealing, or even ablation.

We demonstrate that second and third harmonic generation becomes possible using ultrashort (<180 fs), intense (up to $I\approx 7.8\cdot 10^{16}W/m^{2}$ at ≈78μJ pulse energy) laser pulses and that the photon energy of emission can be tuned without any gap from the near-infrared to ultraviolet spectral range. By analyzing the pulse spectra, intensity dependencies and dispersion features for fundamental, second, and third harmonics, the nonlinear optical nature of the studied phenomenon is unambiguously validated. The harmonic ratio of second- and third-order intensity [19] demonstrates the bulk nature of the underlying photophysical process, i.e., the presence of a ferroelectric polarization within the largest part of the volume of the nanocrystallites. The NLO accessible window shifts as a function of composition due to a shift of the band gap energy. All results are discussed in the framework of NLO applications and in direct comparison to the edge compositions LN and LT.

## 2. Materials and Methods

### 2.1. Mechano-Chemical Synthesis of LNT Nanocrystallites

Nanocrystalline LNT powder samples were synthesized by high-energy planetary ball milling (zirconium oxide balls, 15 h at 600 rpm) of mixtures of Li${\;\;}_{2}$CO${\;\;}_{3}$, Nb${\;\;}_{2}$O${\;\;}_{5}$, and Ta${\;\;}_{2}$O${\;\;}_{5}$ powders, followed by subsequent annealing at temperatures of 700 °C, respectively [18]. Specific compositions of x=0,0.25,0.5,0.75,1 of the system LiNb${\;\;}_{1-x}$Ta${\;\;}_{x}$O${\;\;}_{3}$ were obtained using molar ratios of the components corresponding to the related (calculated) stoichiometry. The crystallite sizes determined via X-ray diffractometry are summarized in Table 1. Additionally, the weighted mean particle sizes <*d*${\;\;}_{\mathrm{DLS}}$> determined by means of dynamic light scattering (DLS) with a Malvern Zetasizer Nano ZSP, as well as the corresponding 5th and 95th percentile ranges of the size distribution, are added.

We would like to note that the DLS results reveal larger size values compared to XRD, which can be attributed to the agglomeration of the LNT nanocrystallites, as already indicated in the transmission electron microscopy images of Ref. [18]. From the viewpoint of the measurement principle, it is a result of the pure optical nature of the DLS technique, determining the hydrodynamical radius based on light scattering. A detailed description of the synthesis procedure, including the description of the (incomplete) intermediate state before annealing, the structural evolution of LNT nanocrystallites and parasitic phases after thermal treatment (five hours at 700 °C), as well as characterization by means of thermogravimetric analysis and Raman spectroscopy of the as-synthesized LNT powders to validate the adjusted compositions, is given in Ref. [18].

### 2.2. Pellet Preparation

**Figure 1:**
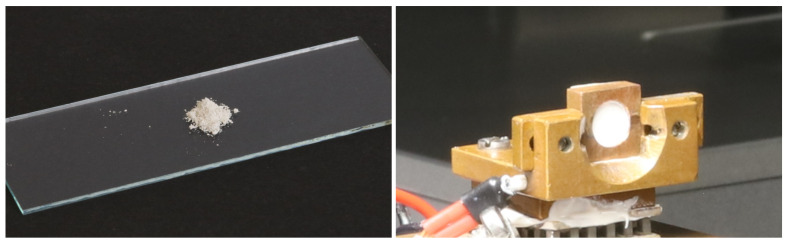
(**Left**): Photo of the loose, as-synthesized LNT nanocrystallites on a microscope slide. (**Right**): Nanocrystallite LNT pressed into a powder pellet in a custom-made copper holder in accordance with the pellet preparation protocol outlined in Ref. [21]. For temperature stabilization, the holder is in thermal contact with a double-stacked Peltier element with a PID-loop controller and a platinum sensor (wired orange cables).

For the purpose of (non-)linear optical investigation, the loose white-colored powder of the as-synthesized LNT nanocrystallites (cf. Figure 1 (left)) is pressed into a densely packed nanoparticle powder pellet in accordance with the procedure described in Ref. [21]. The preparation protocol aims to produce highly diffusely reflecting and opaque samples, i.e., the powder surface acts as a Lambertian scatterer while light transmission through the sample is negligible. Both conditions can be met by compressing the powder into a solid pellet, as elaborated in [22,23], and are important requisites for subsequent data analysis. In our case, a custom-designed copper holder is used as powder support (cf. Figure 1 (right)), as copper provides sufficient thermal conductivity for temperature stabilization. The temperature of the copper holder is kept constant at T=(20±0.5) °C during the experimental series using a double-stacked Peltier element (Peltron GmbH, Fürth, Germany) driven by a proportional–integral–derivative (PID) controller (Profile GmbH, Pro800 series with current driver and PID module).

### 2.3. Remission Spectroscopy

Remission of the as-prepared LNT samples was measured in the spectral window of 250–1700 nm prior to each nonlinear optical measurement. This was performed to estimate the presence of defects and/or parasitic compositions that significantly contribute to the harmonic generation, but also in order to determine the energy of the band gap of all compositions. The latter defines the accessible spectral window for second and third harmonic generation. A commercial two-beam spectrophotometer (Shimadzu Europa GmbH, Duisburg, Germany, UV-3600) equipped with a custom-designed optomechanical module for absolute reflection measurement was used. In detail, an optical system of plane and parabolic ultraviolet-enhanced aluminum mirrors (MPD139-F01, Thorlabs, Newton, NJ, USA) served to direct the probe light as well as to collect the diffuse reflected light from the sample in a reflection geometry (angle of incidence ∼35°). The 1” diameter of the collecting mirror enabled detection within a sufficiently large apex angle well-tailored to the measurement sensitivity of the detection system of the spectrometer. A powder pellet sample of magnesium oxide (MgO) was used as the reference remission sample. MgO features almost unitary reflectance properties within the investigated ultraviolet–visible–near-infrared (UV–VIS–NIR) spectral range of our present study [24,25].

### 2.4. Nonlinear Diffuse Femtosecond Pulse Reflectometry

Characterization of the second- and third-order nonlinearity of the as-synthesized LNT nanocrystallites is performed by means of nonlinear diffuse femtosecond pulse reflectometry, originally introduced by Kijatkin et al. [19]. The method was first demonstrated with Yb-doped LiNbO${\;\;}_{3}$ nanoparticles in reference to TiO${\;\;}_{2}$ powder pellets as an example, and represents a useful tool for the unambiguous characterization of polar and non-polar point symmetry groups of insulating nanoparticles. It was shown that the method is sensitive to particles in the upper layers of the surface, as the penetration depth of the pulse into the powder sample is limited to a few tens of microns. At the same time, it is independent of the light polarization, as the incident polarization state is lost after multiple scattering events [21,26]. In our case, the method is used to demonstrate gap-free emission of SHG and THG, as well as to validate the harmonic nature of the generated signals in LNT nanocrystallites.

The optical technique is based on a regeneratively amplified fs-laser system (Light Conversion, Vilnius, Lithuania, model: Pharos) operating at a central wavelength of 1030 nm (repetition rate: 50 kHz, pulse energy: 400 μJ, average power $\bar{P}=20\;W$, pulse duration $\tau_{\mathrm{pulse}}\;\approx \;240$ fs). This laser serves to pump an optical parametric amplifier (OPA, Light Conversion, model: Orpheus-F) that enables spectral tunability at the output in the range of 650–2500 nm ($\tau_{\mathrm{pulse}}<180$ fs after compression). Intensity-dependent measurements were performed with a sub-50 fs-pulse, high-energy (E${\;\;}_{\mathrm{pulse}}$ = 6 mJ) laser system (Coherent Inc., Saxonburg, PA, USA, model: Astrella) operating at a central wavelength of 800 nm (repetition rate: 1 kHz) that pumps an OPA (Light Conversion, model: TOPAS Prime) with an average power of $\bar{P}=1.2\;W$. In this case, the OPA is kept at a fixed wavelength of 1400 nm (pulse duration: $\tau_{\mathrm{pulse}}\approx 50$ fs), i.e., a wavelength that is very close to the maximum energy of ∼200 μJ of the OPA’s pulse energy spectrum.

The pulse energy used for diffuse reflectometry is adjusted from 1 to 100% by means of a continuously variable neutral density filter wheel (NDC-25C-2, Thorlabs). Autocorrelators (APE Angewandte Physik und Elektronik GmbH, Berlin, Germany, model: PulseCheck and Light Conversion, model: GECO) served for the determination of the pulse duration at the position of the sample for all wavelengths (in the sub-180 fs and sub-60 fs time range for Pharos and Astrella, respectively; a Gaussian pulse-shape is assumed for the analysis of the temporal profile). The pulses are deflected by a set of mirrors and focused onto the LNT pellets by an off-axis parabolic mirror (MPD169-P01, Thorlabs) at normal incidence. Intensity and spectral distribution of the diffusive (non-specular) reflected light is collected at an angle of ≈20° with respect to the incident pulse path by a single-mode optical fiber and fed into a spectrometer system. A fiber spectrometer of the model QEPro (OceanInsight Inc., Orlando, FL, USA) is used for detection in the visible spectral range from 280 to 1050 nm with a shortpass filter (FESH750, Thorlabs) to suppress the fundamental and the model SR-90 (TOPAG Lasertechnik GmbH, Darmstadt, Germany) for detection in the near-infrared spectral range from 990 to 1720 nm. The same spectrometers were used for spectral inspection of the incident pulses.

For photographic imaging of the harmonic remission of the LNT powder pellets, a digital camera (EOS80D, Canon, Tokyo, Japan) is used. A short-pass filter with a 50% cut-off wavelength of ≈700 nm (KG3, Schott, Mainz, Germany) is mounted in front of the objective of the camera to suppress the intense infrared fundamental radiation, thereby preventing an oversaturation of the image sensor.

## 3. Sample Characterization

### Remission Spectroscopy

**Figure 2:**
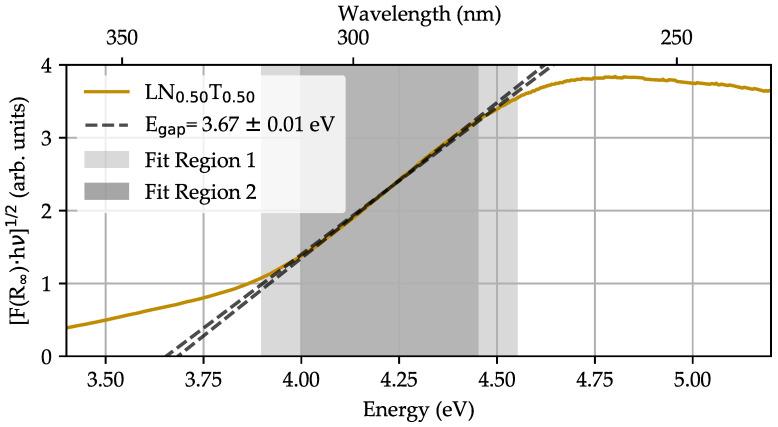
Tauc plot of the remission in the blue-ultraviolet energy spectrum for LN${\;\;}_{0.50}$T${\;\;}_{0.50}$. Linear functions (dashed lines) are fitted to the experimental dataset (ochre line) for two distinct fit regions in the range from 4.0 to 4.45 eV and 3.9 to 4.55 eV. The zero crossing point, averaged from both fits, is located at a band gap energy of $E_{\mathrm{gap}}=\left(3.67\pm 0.01\right)$ eV. An indirect allowed transition is assumed in the analysis.

Remission spectra $R_{\infty }\left(\lambda \right)$ were repeatedly determined for all LNT compositions in reference to the reflectance of a white standard (MgO) in the spectral range from 250 to 1700 nm following a three-fold protocol: measurement MgO, measurement LNT, measurement MgO, with the goal to optimize the signal-to-noise ratio (SNR). In comparison to MgO, the overall diffuse reflectance $R_{\infty }\left(\lambda \right)$ of the LNT powder pellets is attenuated by up to 20% for VIS wavelengths and even increased by up to ≈25% for NIR wavelengths, thus resembling a broad optical window well-suited for NLO studies. To estimate the maximum photon energy for frequency conversion, i.e., to determine the energy of the optical band gap E${\;\;}_{\mathrm{gap}}$, the Kubelka–Munk function [27] $F\left(R_{\infty }\right)={\left(1-R_{\infty }\right)}^{2}/2R_{\infty }$ is used to plot ${\left[F\left(R_{\infty }\right)\cdot h\nu \right]}^{1/2}$ as a function of hν, commonly known as a Tauc plot. Figure 2 shows the result in the blue-ultraviolet spectral region between 3.4 and 5.2 eV with LN${\;\;}_{0.50}$T${\;\;}_{0.50}$ as an example.

An indirect, allowed transition is assumed for LNT [28] and the band gap energy is expected in the inspected spectral region from 3.5 to 5.0 eV according to the corresponding values of the edge compositions ($E_{\mathrm{gap}}\left(\mathrm{LN}\right)=3.78$ eV [29,30,31] and $E_{\mathrm{gap}}\left(\mathrm{LT}\right)$ = 4.5–4.7 eV [32,33,34]). The band gap can be determined from the Tauc plots via the relation ${\left[F\left(R_{\infty }\right)\cdot h\nu \right]}^{1/2}=\mathrm{const}.\cdot \left(h\nu -E_{\mathrm{gap}}\right)$ [35] and by fitting a linear function to the linear region of the plot. The value $E_{\mathrm{gap}}$ corresponds to the zero crossing of the linear fit function. The Tauc plot, linear fitting, and determined band gap energy are depicted in Figure 2, exemplary for the composition LN${\;\;}_{0.50}$T${\;\;}_{0.50}$. The data show a well-defined linear behavior in the range from 4.0 to 4.45 eV, which is used for the linear fitting procedure and results in a band gap energy of $E_{\mathrm{gap}}=\left(3.67\pm 0.01\right)$ eV. The error is estimated by changing the function’s slope within an acceptable deviation from the dataset and taking the data accuracy for $R_{\infty }$ into account. We have applied this procedure to all LNT compositions x=0,0.25,0.5,0.75,1.0 and were able to find a linear slope in a similar spectral region in the datasets. The determined values for the indirect band gap energies and corresponding wavelengths $\lambda_{\mathrm{gap}}$ are summarized in Table 2.

We would like to note that the analysis of the data revealed indications for the presence of impurities at a very low concentration in the samples. In particular, a slight remission decrease, i.e., an absorption increase, is observed in the low-energy region of up to ∼3.8 eV. In addition to impurities at the ppm level, it may also be attributed to parasitic phases (Li(Nb/Ta)${\;\;}_{3}$O${\;\;}_{8}$) from the synthesis of LNT [18]. However, due to the low amplitude, we can exclude a significant influence of the nonlinear optical response under study.

## 4. Nonlinear Diffuse fs-Pulse Reflectometry

### 4.1. Harmonic Generation

**Figure 3:**
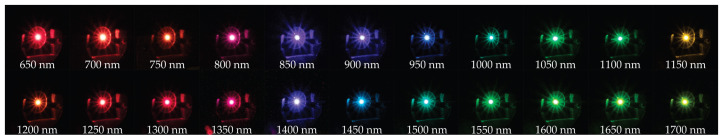
Digital images (spectral detection window of the digital camera ≈ 350–700 nm) of diffuse remission of LN${\;\;}_{0.50}$T${\;\;}_{0.50}$ nanoparticles under exposure to a train of fs-pulses tuned from 650 to 1700nm in steps of 50 nm. The respective fundamental wavelengths are below each image.

The generation of harmonic emission of LNT nanopowders upon exposure to a train of ultrashort intense laser pulses is validated by visual inspection as a starting point for nonlinear diffuse fs-pulse reflectometry (cf. Figure 3). For this purpose, fundamental pulses at selected wavelengths within the spectral range of 650–1700 nm are used for exposure of the LNT pellets (repetition rate of 50 kHz, average power of $\bar{P}$ = 0.05–0.3 W), while the diffuse reflected light is captured by a digital camera. The image series depicted in Figure 3 is obtained exemplarily for LN${\;\;}_{0.50}$T${\;\;}_{0.50}$ nanoparticles using one and the same sample for all photographs. According to its wavelength limit of $\lambda_{\mathrm{Fund},\min}^{\mathrm{SHG}}$ (cf. Table 2), the fundamental pulse scan was started at λ=650 nm in steps of 50nm up to a maximum of λ=1700 nm, thus yielding a total of 22 images (exposure time: 0.2–20 s ≡ $10^{3}$–$10^{6}$ pulses).

All digital images show light emission of different colors, with an intense light spot in the central region at the point of laser incidence. The bright spot is surrounded by a scattering corona that is further reflected/scattered by parts of the copper sample holder. The colors cover the entire rainbow spectrum from ultraviolet, blue, green, yellow, orange, red up to near-infrared.

### 4.2. Diffuse fs-Pulse Remission Spectra

To complement the visual examination, the diffuse fs-pulse remission is analyzed spectroscopically as a function of the fundamental wavelength in the range 1200 nm $<\lambda_{\mathrm{Fund}}<2000$ nm in steps of 10 nm and with a spectral resolution of up to ∼1.7 nm. The wavelength range for $\lambda_{\mathrm{Fund}}$ is chosen in accordance with the spectral detection window of the spectrometer, taking into account the expected wavelength limits $\lambda_{\mathrm{Fund},\min}^{\mathrm{SHG},\mathrm{THG}}$ for second and third harmonic generation, as listed in Table 2. We note that the short-pass filter is not used in this case. A time-averaged power in the range of $\bar{P}\approx$ 0.05–0.3 W was adjusted at all wavelengths (the average power gradually declines down to 50 mW, due to the diminishing conversion efficiency of the OPA towards the upper NIR spectral range). In this way, the integration time for spectra detection of 9 ms could remain constant and the dynamic range of 18 Bit of the A/D converter was utilized to a maximum.

**Figure 4:**
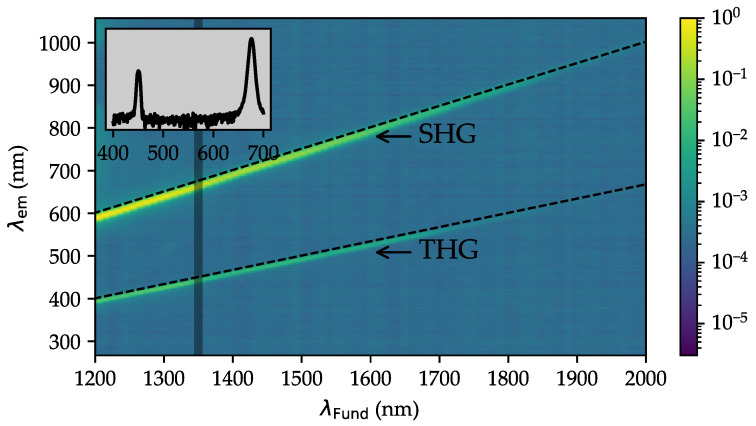
Two-dimensional colormap measurement of LN${\;\;}_{0.50}$T${\;\;}_{0.50}$. The emission spectra are shown as a function of the fundamental wavelength with a color grade of the intensity, increasing from dark blue to light yellow colors on a log scale. The inset shows the remission spectrum of the second and third harmonic at a fundamental wavelength of 1350 nm on a logarithmic scale.

Figure 4 shows the obtained set of 80 spectra for the wavelength dependence of the remission intensity $I(\lambda_{\mathrm{em}})$ (averaged over 100 measurements, normalized to the dataset at 1200 nm) as a function of the fundamental wavelength $\lambda_{\mathrm{Fund}}$, exemplary for the LN${\;\;}_{0.50}$T${\;\;}_{0.50}$ sample. For the sake of clarity, it is displayed as a 2D color plot with the remission intensity depicted as a color gradient.

The plot reveals two distinct spectral traces (green/yellow regions) in front of a background of vanishing signal (blue area). The upper, broader trace starts to emit at about $\lambda_{\mathrm{em}}=600$ nm ($\lambda_{\mathrm{Fund}}=1200$ nm) and rises up to $\lambda_{\mathrm{em}}=1000$ nm ($\lambda_{\mathrm{Fund}}=2000$ nm). Accordingly, the lower trace shows an emission shift for $\lambda_{\mathrm{em}}$ from 400 nm to 667 nm. It is noteworthy that the emissions of the two traces are detected simultaneously in the individual spectra, i.e., both emission processes appear under the same pumping conditions of the fundamental pulse sequence (cf. inset of Figure 4). As a second prominent feature, the trace intensity develops continuously, so that the intensity difference in the trace maxima between two subsequent traces is much below 10% and so that signals related to both traces are found in all spectra up to $\lambda_{\mathrm{Fund}}\approx 1800$ nm with sufficient SNR. In the range up to 2000 nm, the lower trace disappears in the detector’s noise. The third, most important property of the two traces is an obvious linear relation between the emission ($\lambda_{\mathrm{em}}$) and the fundamental wavelengths ($\lambda_{\mathrm{Fund}}$). In both cases, fitting of the traces with a linear function of type $\lambda_{\mathrm{em}}=n\cdot \lambda_{\mathrm{Fund}}$ was possible (dotted lines in Figure 4), yielding slope values of n=0.50±0.01 (upper trace) and n=0.33±0.01 (lower trace). The traces thus resemble wavelength conversion to half or a third of the fundamental wavelength, i.e., corresponding to the doubled and tripled frequency, a process that is commonly referred to as second (SHG) and third (THG) harmonic generation, respectively. From the analysis of corresponding spectra series detected for LNT nanopowders with x=0,0.25,0.5,0.75,1.0, this finding was validated in all compositions under study.

### 4.3. Wavelength Dependence of the Harmonic Intensities

**Figure 5:**
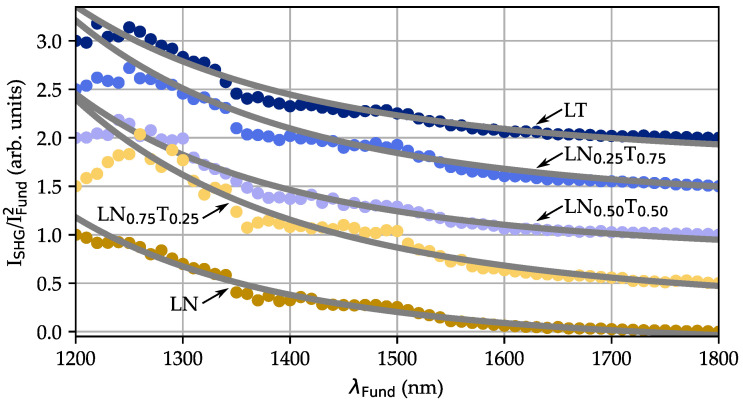
Intensity dependence of the second harmonic signal (arbitrary units) as a function of the fundamental wavelength $\lambda_{\mathrm{Fund}}$ for all LNT compositions with x=0,0.25,0.5,0.75,1.0. The signal data were deduced from the individual emission spectra (cf. upper trace in Figure 4) and normalized to the square of the fundamental pulse peak intensity (details in the text). For the sake of clarity, the five plots were mutually displaced to each other along the signal axis. The dotted lines represent fits of functions of type $1/{\left(\lambda -\lambda_{\mathrm{gap}}\right)}^{m}$ to the datasets (cf. discussion).

The dependency of the second and third harmonic intensities as a function of the fundamental wavelength $\lambda_{\mathrm{Fund}}$ has been analyzed in more detail and is plotted in a comparative manner for all compositions in Figure 5. As an example, the wavelength dependence of the normalized emission signal related to the second harmonic generation $I\left(\lambda_{\mathrm{em}}^{\mathrm{SHG}}\right)/I^{2}\left(\lambda_{\mathrm{Fund}}\right)$ is shown; it is deduced from the spectra following the procedure described as follows. $I\left(\lambda_{\mathrm{em}}^{\mathrm{SHG}}\right)$ was determined individually from the emission spectra for each wavelength $\lambda_{\mathrm{Fund}}$ from the integral area of a fit with a Gaussian function to the related SHG (and THG) signal peaks and normalized to the square of the pulse peak intensity of the fundamental pulse $I^{2}\left(\lambda_{\mathrm{Fund}}\right)$. The latter was determined by measuring the average power and the estimated area of exposure for each wavelength.

All five datasets show qualitatively comparable behavior; the signal emission intensity decreases with a nonlinear trend from 1200 nm to 1800 nm by more than 90%. For a quantitative comparison, the function $I_{\mathrm{SHG}}\propto \;1/{\left(\lambda -\lambda_{\mathrm{gap}}\right)}^{m}$ was fitted to the datasets, yielding values m(x=0)=(4.29±0.01), m(x=0.25)=(4.19±0.01), m(x=0.5)=(4.27±0.01), m(x=0.75)=(4.22±0.01), and m(x=1)=(4.22±0.01).

### 4.4. Intensity Dependencies of Harmonic Emission

**Figure 6:**
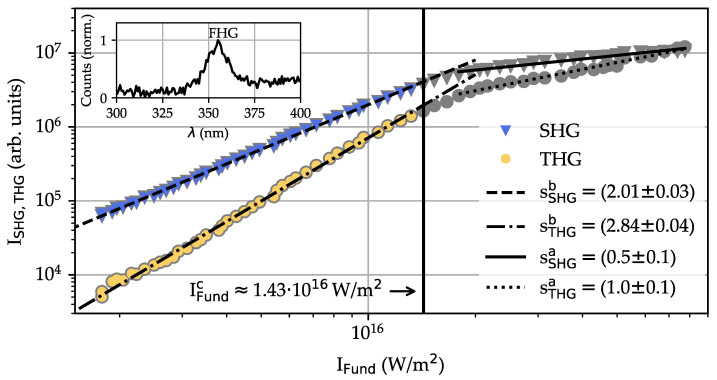
Intensity dependence of the second- and third-order harmonic signals (triangles and circles, respectively) as a function of the intensity of the fundamental pulse in a double-logarithmic plot. As an example, data are shown for the sample LN${\;\;}_{0.50}$T${\;\;}_{0.50}$ at a fundamental wavelength of 1400nm. The emission intensities at 700 nm (SHG) and 467 nm (THG) show a pronounced increase, with a significant change in both slopes at about $1.43\cdot 10^{16}$ W/m${\;\;}^{2}$. A linear function is fitted to both datasets (dotted black lines) for both slopes. The values of the derived slopes n${\;\;}_{\mathrm{SHG}}$, n${\;\;}_{\mathrm{THG}}$ of the fit functions are given in the inset. A spectrum of the fourth harmonic generation (FHG) is added to the figure as an inset.

Figure 6 shows the intensities of the second- and third-order emission signals as a function of the intensity of the fundamental pulse for the fundamental wavelength $\lambda_{\mathrm{Fund}}=1400$ nm in the range from $I_{\mathrm{Fund}}\left(1400\right)\approx 1.8\cdot 10^{15}$ W/m${\;\;}^{2}$ to $I_{\mathrm{Fund}}\left(1400\right)\approx 7.8\cdot 10^{16}$ W/m${\;\;}^{2}$. The harmonic signal intensities were determined from individual emission spectra, as described above, and thus are presented in arbitrary units. The pulse peak intensity (W/m${\;\;}^{2}$) of the incident pulse was determined by measuring its mean average power and pulse duration (repetition rate: 1 kHz). The pulse duration remained at a constant mean value of $\tau_{p}=\left(48\pm 5\right)$ fs for the entire range of intensities. The choice of 1400 nm with second- and third-order signals peaking at 700 nm and 467 nm, respectively, enabled spectral detection that is completely covered by the UV/VIS fiber spectrometer. As a result, a sufficient SNR > 1 for the second- and third-order signals was accessible. We note that a double-logarithmic plot is used in Figure 6.

The evaluation of the data shows an overall significant increase in both second- (blue/gray triangles) and third-order (yellow/gray circles) signal intensities as a function of the fundamental intensity, with a total increase of about two (SHG) and four (THG) orders of magnitude. A change in the functional dependencies appears for both harmonic signals at an intensity of $I_{\mathrm{Fund}}^{c}\approx 1.43\cdot 10^{16}$ W/m${\;\;}^{2}$ (at a corresponding pulse energy of E${\;\;}_{p}\approx 14.3\;\mu$J). Below and above this prominent point, linear dependencies can be assumed, with significantly different slopes for the second and third harmonics. Accordingly, power functions of the type $I_{\left(\mathrm{SHG},\mathrm{THG}\right)}=c\cdot I_{\mathrm{Fund}}^{s_{\left(\mathrm{SHG},\mathrm{THG}\right)}^{a,b}}$ were fitted to the data points separately for both intensity ranges (the superscripts a,b denote above and below $I_{\mathrm{Fund}}^{c}$) and harmonic signals. Qualitatively, a similar intensity behavior is found for all five compositions with x=0,0.25,0.5,0.75,1.0 and was analyzed in all datasets accordingly. The results for the intensity range below $I_{\mathrm{Fund}}^{c}$ are listed in Table 3 and reveal slopes of $s_{\mathrm{SHG}}^{b}\approx 2$ and of $s_{\mathrm{SHG}}^{b}\approx 3$ for all compositions under study.

For intensities above $I_{\mathrm{Fund}}^{c}$, the slopes reduce to values $s_{\mathrm{SHG},\mathrm{THG}}^{a}\leq 1$. We would like to add that the spectral features were inspected as a function of time over a duration of one hour. No changes in the signals (amplitude and spectral position) were observed, so that light-induced alterations of structure and/or composition and/or clustering/agglomeration as a result of the intense laser exposure can be excluded.

### 4.5. Harmonic Ratio

**Figure 7:**
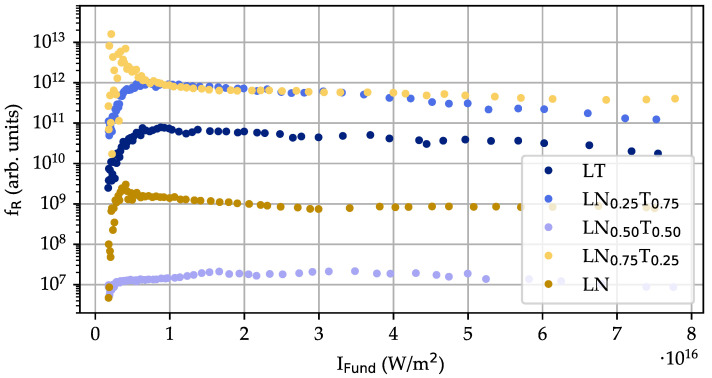
Harmonic ratio as a function of the fundamental peak intensity for all LNT compositions under study.

The intensity dependencies of second- and third-order harmonics were further used to determine the harmonic ratio [19]:

(1) $$\begin{array}{c} f_{R}=\frac{I_{2\omega }^{3}}{I_{3\omega }^{2}}=\frac{I_{\mathrm{SHG}}^{3}}{I_{\mathrm{THG}}^{2}} \end{array}$$

as a function of the intensity of the fundamental pulse, i.e., $f_{R}\left(I_{\mathrm{Fund}}\right)$ was determined for all LNT compositions (see Figure 7).

The dataset shows a pronounced increase at the lowest intensities that changes to a saturation value of $f_{R}$ for intensities above $I_{\mathrm{Fund}}\approx 5\cdot 10^{15}$ W/m${\;\;}^{2}$, i.e., $f_{R}\left(I_{\mathrm{Fund}}\right)$ = $f_{R}$ = const. Remarkably, the saturation behavior remains almost unaffected by the fact that the underlying intensity dependencies of SHG and THG show a change in the slope at $I_{\mathrm{Fund}}^{c}\approx 1.43\cdot 10^{16}$ W/m${\;\;}^{2}$ (cf. Figure 6). Furthermore, the saturation values of $f_{R}$ differ by more than five orders of magnitude for the studied compositions (from $f_{R}\left({\mathrm{LN}}_{0.5}T_{0.5}\right)\approx 10^{7}$ up to $f_{R}\left({\mathrm{LN}}_{0.25}T_{0.75}\right)\approx 10^{12}$), though no trend towards a composition dependence $f_{R}\left(x\right)$ is found. Overall, $f_{R}\gg 1$ is found for all samples without any doubt.

### 4.6. Bandwidths of Harmonic Emission

**Figure 8:**
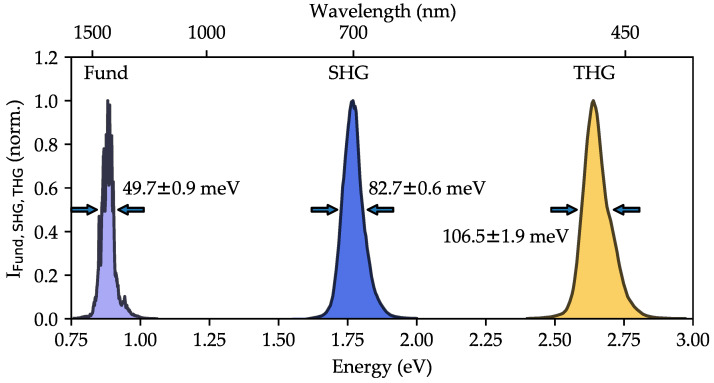
Spectral fingerprints of the fundamental, SHG, and THG emission in LN${\;\;}_{0.50}$T${\;\;}_{0.50}$. The fundamental, second, and third harmonic are normalized to their respective maximum to increase visibility. Two opposing arrows indicate the FWHM of each spectral signature.

Figure 8 shows a comparison of the spectral intensity profiles of the incident fundamental pulse with those of the second- and third-order emissions, as measured in one of the sets of spectra by nonlinear diffuse fs-pulse reflectometry. Exemplarily, the data are shown for the LN${\;\;}_{0.50}$T${\;\;}_{0.50}$ sample and for a fundamental wavelength of 1400 nm at an intensity of $I_{\mathrm{Fund}}\left(1400\right)\approx 6\cdot 10^{16}$ W/m${\;\;}^{2}$. In this case, SHG appears at 700 nm and THG at 467 nm. For reasons of proper data analysis, the signal intensities (arbitrary units) are normalized to the intensity value of the respective peak maximum and are plotted as a function of photon energy.

The profiles show a slight asymmetry in the spectral domain, with the tendency of a longer tail to larger photon energies as a result of the spectral (complex) pulse profiles at the output of the OPA. In this contribution, a focus of the spectra analysis lies on their spectral bandwidths, in particular, the comparison between the full widths at half maxima of the signals. As a general trend, a pronounced broadening of the pulses with increasing harmonic order is found in the domain of photon energies with a clear series FWHM${\;\;}_{\mathrm{Fund}}<$ FWHM${\;\;}_{\mathrm{SHG}}<$ FWHM${\;\;}_{\mathrm{THG}}$. For a quantitative comparison, the standard deviations σ of Gaussian fits centered at 1400 nm, 700 nm, and 467 nm were used to determine the FWHM-values via FWHM$=2\sqrt{2\ln2}\sigma$. As a result, the spectral bandwidths are determined as (78.7±2.7 nm, 49.7±0.9 meV) for the fundamental (1400 nm, 0.89 eV), (32.7±0.5 nm, 82.7±0.6 meV) for the second harmonic (700 nm, 1.77 eV), and (18.7±0.7 nm, 106.5±1.9 meV) for the third harmonic (466 nm, 2.66 eV). The relative ratio of the FWHM between fundamental and harmonics can thus be expressed as 1:1.66 for SHG and 1:2.14 for THG.

## 5. Discussion

### 5.1. Application of Nonlinear Diffuse fs-Pulse Reflectometry to LNT Nanoparticle Pellets

The example of mechanochemically synthesized LNT nanoparticles shows once again that the interaction of (ultra-)short laser pulses with polar nano- and/or microparticles, as originally introduced in the work of Kurtz and Perry [20], is a powerful tool for the characterization of novel nonlinear optical materials. The extension of the method by very short, intense femtosecond light pulses, tunable in photon energy, as introduced by Kijatkin et al. [19], leads to the possibility of inspection of harmonics at elevated intensities (here, up to a few $10^{16}$ W/m${\;\;}^{2}$), thus resulting in the appearance of higher-order harmonics. It is a result of the technical development of (commercially available) high-energy fs-laser systems based on the inventions of chirped pulse amplification (CPA-technology) [36] and femtosecond technology of the second and third generations [37]. At the same time, laser-induced alterations of the LNT crystallites (e.g., via thermally induced melting, agglomeration, dielectric breakdown, or any other phenomena affecting the crystallite structures) can be neglected. Even with continuous fs-pulse irradiation over a period of one hour, neither changes in the emission intensities nor the spectral position of the emissions (within the experimental error) could be detected. We note here that the average laser power density $\bar{P}/A$ with the area of exposure A was kept well below the values of thermo-optical breakdown reported for LN (in the order of 10${\;\;}^{4}$ W/cm${\;\;}^{2}$[38]). Furthermore, the pulse energy density $E_{\mathrm{pulse}}/A$ did not exceed values reported for dielectric breakdown and multiphoton avalanche ionization in LN [39] (in the order of 0.1 J/cm${\;\;}^{2}$ for uncoated LN [40]).

### 5.2. Characterization of LNT Nanocrystallites from the (Non-)linear Optical Perspective

Harmonic generation and its gap-free tuning particularly require the possibility to match the phase velocities between fundamental and harmonic waves along the interaction length over the entire accessed wavelength spectrum. A characteristic measure is the nonlinear optical coherence length $l_{\mathrm{coh}}$[41], i.e., phase-matching in the LNT nanocrystallites requires that the condition $d_{\mathrm{size}}\ll l_{\mathrm{coh}}$ be fulfilled. In our case, we find, for example, $l_{\mathrm{coh}}$(SHG) ≈6.4μm and $l_{\mathrm{coh}}$(THG) ≈1.58μm using the index of refraction of LN (n(1400nm)=2.2164, n(700nm)=2.2711, and n(467nm)=2.3641 [42]). Taking into account the fact that LNT crystallite sizes are far below 500 nm (cf. Table 1), the condition $d_{\mathrm{size}}\ll l_{\mathrm{coh}}$ is fulfilled for SHG and THG without any doubt.

The results of the band gap energies obtained by remission spectroscopy give important hints for the accessible spectral range for harmonic generation. In particular, harmonic emission will be strongly damped with increasing linear absorption, following Beer’s law $I_{\mathrm{SHG},\mathrm{THG}}\cdot \exp\left(-\alpha \cdot d\right)$. If the band edge is used as the limit value, the smallest fundamental wavelength is determined by $\lambda_{\mathrm{Fund},\min}^{\mathrm{SHG}}=2\cdot \lambda_{\mathrm{gap}}$ for SHG and $\lambda_{\mathrm{Fund},\min}^{\mathrm{THG}}=3\cdot \lambda_{\mathrm{gap}}$ for THG. According to Table 2, the band gap energies vary as a function of composition, resulting in varying accessible spectral ranges for harmonic generation: between 548 and 676 nm for SHG and between 822 and 1014 nm for THG. This corresponds to a difference of about ∼125 nm and ∼190 nm for SHG and THG, respectively. At the edge compositions of LN (3.76 ± 0.02 eV) and LT (4.53 ± 0.01 eV), the estimated band gaps coincide with values of the respective bulk material [29,30,31,32,33,34]. We would like to highlight here the band gap energy of LN${\;\;}_{0.50}$T${\;\;}_{0.50}$ of 3.67 ± 0.01 eV, which is found to be below those for LN and LT. This means that the band gap can be shifted by composition in LNT to lower as well as higher energies in comparison to the reference system LN.

### 5.3. Harmonic Generation

The experimentally determined emission properties of the LNT nanoparticle pellets, in particular the observed color emissions, the wavelength relations between fundamental and harmonic emissions, the intensity dependencies of the harmonics, and the mutual relations of the spectral bandwidths, inevitably point to the presence of second and third harmonic generation under fs-pulse exposure and will be discussed individually in the framework of expectations from nonlinear optical theory as follows.

The observed emission features under fs-pulse exposure with wavelengths from 650 to 1700 nm depicted in Figure 3 reveal a broad color spectrum with an individual sequence: red-blue-green-yellow-red-blue-green-yellow. Color analysis requires consideration of the superposition of diffuse reflected light of the fundamental and the harmonic emission of second and/or third order, as well as the spectral sensitivity of the sensor chip of the camera (Si-based charged coupled display with sensitivity in the visible/near-infrared spectral range from 350 to 1100 nm) clipped to the spectral range of 350–700 nm by the short-pass filter. At the lowest fundamental wavelengths of 650, 700, and 750 nm, only the (pure) red color of the diffuse reflected fundamental is detected. At a fundamental of 800 nm and above, the second harmonic emission is generated simultaneously with the diffuse red light of the fundamental pulse, thus resulting in a violet color emission. At larger wavelengths, the fundamental is sufficiently blocked, and the second harmonic emission starts to dominate the visual impression of remission. It shows a continuous change from blue over turquoise, to green, to yellow, to orange, and finally to red at a fundamental wavelength of 1300 nm. The transition from detecting the second to third harmonic generation takes place in the wavelength range from 1300 to 1400 nm, with a change from red to blue. Here, the third harmonic generation starts to match the visible spectral range of detection and constitutes the primary color until green/yellow emission at the end of the inspected spectral range (λ = 1700 nm).

The interplay of simultaneous second and third harmonic emission is validated by the spectral inspection of the emission shown in the 2D color map of Figure 4. However, due to the larger dynamic range and increased sensitivity of the spectroscopic detection system, as well as the broader spectral range of detection from 280 to 1050 nm, SHG and THG signals are detected all over the (near-)infrared spectrum of the fundamental pulse between 1200 and 2000 nm. Furthermore, the 2D plot enables a first impression of the relative intensities between second- and third-order emission, showing a dominant contribution of SHG at all wavelengths according to the color coding. The spectrum obtained at 1350 nm (see inset of Figure 4) supports this impression, exemplifying the dominant SHG-color contribution seen in Figure 3 at fundamental wavelengths up to ∼1400 nm. The 2D color plot is particularly helpful for the analysis of the wavelength relations between fundamental and emission wavelengths, which must follow a linear behavior $\lambda_{\mathrm{SHG},\mathrm{THG}}=n\cdot \lambda_{\mathrm{Fund}}$, with n=1/2 for the case of SHG and n=1/3 for the case of THG, according to theory. The determined slope parameters of n=(0.50±0.01) and n=(0.33±0.01) for the upper and lower traces, respectively, are found in very good accordance with these expectations, thus representing a strong argument for the presence of harmonic generation in the studied LNT nanoparticles.

The intensity dispersion, exemplarily shown for SHG in Figure 5, demonstrates that the tuning of harmonic generation is possible in the range from 1200 to 1800 nm without any gap. Furthermore, the intensity shows a pronounced, nonlinear increase from long to short fundamental wavelengths for all LNT compositions. For a proper analysis of the dispersion behavior, the SHG intensity data are normalized to the square of the fundamental intensities according to the theoretical dependence for the SHG efficiency in nonlinear optical theory [1]: $I_{\mathrm{SHG}}\propto I_{\mathrm{Fund}}^{2}\cdot \chi^{\left(2\right)}\left(\lambda_{\mathrm{SHG}},\lambda_{\mathrm{Fund}}\right)/n_{\mathrm{SHG}}\cdot n_{\mathrm{Fund}}^{2}\cdot \lambda_{\mathrm{Fund}}^{2}$ (assuming slow varying amplitudes, i.e., no pump beam depletion, a negligible phase mismatch, and energy transfer in the crystallite bulk). Obviously, dispersion of the SHG signal is due to the interplay of the wavelength dependencies of the second-order susceptibility tensor, the indices of refraction for fundamental and harmonic waves (at least described by the Cauchy relation [43]: $I\left(\lambda_{\mathrm{em}}^{\mathrm{SHG}}\right)=A+B/{\left(\lambda -\lambda_{0}\right)}^{2}$ with a constant fitting parameter A and an offset wavelength $\lambda_{0}$), as well as the fundamental wavelength itself. For LN and LT, the dispersion of the index of refraction can be effectively modeled by the Sellmeier equation [44] and parameters published, e.g., in Refs. [42,45]. The susceptibility $\chi^{\left(2\right)}\left(\lambda \right)$ is expected to reveal a similar dispersion, if considered as an upcoming element of the higher-order series extension of the polarization [1]. This assumption is well-supported by the work of Xue [17] showing that the basic concept of anharmonicities of induced dipole oscillations is well-justified for LNT. Taking all individual dispersion features into account, an overall wavelength dependence for $I_{\mathrm{SHG}}\propto 1/{\left(\lambda -\lambda_{\mathrm{gap}}\right)}^{m}$ with m≫2 can be expected that accords well with the result of m=(4.24±0.05) from the fitting procedure to the experimental dataset (see Figure 5). A more detailed analysis of the dispersion and, particularly, of the nonlinear exponent m fails due to the lack of more precise data on the dispersion of $n_{\mathrm{SHG},\mathrm{THG}}\left(\lambda \right),\chi \left(\lambda \right)$ for LNT, but also due to the spectrally limited window of experimental data. However, a reasonable coincidence between the data and the expectations from theory can already be concluded.

We would like to add that the ability to draw quantitative conclusions about the wavelength dependence is also severely limited by the complex scattering behavior of the nanoparticle ensemble. As already modeled in Ref. [21], the scattering behavior depends on the particle size, the wavelength, the degree of particle agglomeration, as well as the surface morphology of the powder pellet, in addition to the dispersion of the index of refraction. It is also necessary to consider the optical transfer function of the detection system, i.e., the dispersive properties of the reflection/transmission losses of the grating, the optical fiber, and the sensor array. As a result, theoretical, particularly numerical, modeling is required to provide comprehensive information on the various dispersion contributions to the harmonic signals.

At the same time, it can be concluded from the theory that—for a fixed wavelength—the intensity dependencies $I_{\left(\mathrm{SHG},\mathrm{THG}\right)}\left(I_{\mathrm{Fund}}\right)$ can be analyzed by the simplified relations $I_{\mathrm{SHG}}\propto I_{\mathrm{Fund}}^{2}$ and $I_{\mathrm{THG}}\propto I_{\mathrm{Fund}}^{3}$, as demonstrated in Figure 6 for the LNT composition with x=0.5 and $\lambda_{\mathrm{Fund}}=1400$ nm. The quadratic and cubic increases resemble the datasets and the results of the corresponding linear fitting procedures (in the double-logarithmic presentation) up to intensities of $I_{\mathrm{Fund}}^{c}\approx 1.43\cdot 10^{16}$ W/m${\;\;}^{2}$ (cf. Table 3) with respective (composition-)averaged slopes of ${\bar{s}}_{\mathrm{SHG}}≊1.91$ and ${\bar{s}}_{\mathrm{THG}}≊2.83$. Thus, again, the assumption of second and third harmonic generation during exposure to fs-pulse trains is well-justified in all LNT nanoparticles. Beyond $I_{\mathrm{Fund}}^{c}$, however, the intensity dependence shows a pronounced change in the slope, while a linear dependence remains. In both cases, $s_{\mathrm{SHG},\mathrm{THG}}\leq 1$ is determined from the fitting procedure. This behavior may likely be attributed to multiple effects: (i) the appearance of pump beam depletion with substantial magnitude due to the high pulse peak intensity of the fundamental; (ii) the appearance of cascaded frequency conversion effects, such as sum frequency mixing between second harmonic and fundamental pulses that yields third harmonics as well. Such processes particularly appear in the presence of sufficiently high SHG intensities. (iii) The third effect is the conversion into higher harmonic orders, such as fourth and fifth harmonic generation, as experimentally observed in the spectrum depicted in the inset of Figure 6. We note that higher harmonics also require sufficiently large intensities of the fundamental. Furthermore, the wavelengths of higher harmonics may be generated below the band gap wavelengths and, thus, are likely to be reabsorbed completely. All three effects (and maybe further ones) are characterized by different individual intensity dependencies and, if appearing in parallel, will have a strong impact on the overall intensity dependency. Nevertheless, the effect of pump depletion can be assumed to be the dominating factor, as the conversion efficiency for fourth harmonic generation can be considered negligible.

The intensity dependencies for SHG and THG can be effectively applied to determine the harmonic ratio $f_{R}\left(I_{\mathrm{Fund}}\right)$ as a function of the fundamental pulse peak intensity. The harmonic ratio is a reliable indicator for the polarity of the crystal symmetry of the nanoparticles and indicates a polar symmetry if $f_{R}\gg 1$. In our case, $f_{R}$ could be determined in the range of intensities from $5\cdot 10^{15}$–$7.8\cdot 10^{16}$W/m${\;\;}^{2}$ and reveals $f_{R}\gg 10^{6}\gg 1$ for all intensities and all compositions, i.e., a clear indication for the presence of a ferroelectric polarization in the LNT nanoparticles can be concluded. At the same time, a clear trend for the dependence of $f_{R}$ on the composition is not apparent. We note here that it is not necessarily to be expected, as the particle size has a significant effect on the magnitude of the harmonic ratio ($f_{R}\propto d_{\mathrm{size}}^{2}$) as well. The pronounced growth of the harmonic ratio at low intensities is attributed to the limited signal-to-noise ratio of SHG and particularly THG intensities, as discussed in Ref. [19].

Finally, the results for the spectral bandwidths of fundamental, second-, and third-order harmonics are compared with the theoretical expectations from nonlinear optics [46], which predicts a pulse broadening according to FWHM${\;\;}_{n}=\sqrt{n}\cdot$FWHM${\;\;}_{\mathrm{Fund}}$ for pulses with spectral Gaussian profiles, where FWHM${\;\;}_{n}$ indicates the spectral bandwidth of the *n*-th harmonic order. The results of $\sqrt{n}\approx 1.66$ for the SHG peak and $\sqrt{n}\approx 2.14$ for the THG peak are found in acceptable agreement with the theoretical expectations of $\sqrt{2}$ and $\sqrt{3}$, respectively. Deviations from the theoretical expectations can be assigned to deviations of the fs-pulse profiles from the Gaussian profile, as well as to the asymmetric profiles.

## 6. Summary and Conclusions

Summarizing our study, the generation of second and third harmonics and the possibility for gap-free tuning down to emission wavelengths of ≈340 nm is experimentally verified and can be unambiguously assigned to the process of frequency conversion according to the following results: (i) a linear dependence between the wavelengths of fundamental and harmonic expressed by a slope of factor 1/2 (SHG) and 1/3 (THG), (ii) a spectral dependence of the harmonic intensity showing a nonlinear increase for decreasing wavelengths, (iii) a nonlinear intensity dependence with a quadratic (SHG) and cubic (THG) increase as a function of the fundamental intensity, and (iv) a spectral broadening of the generated pulse profiles by a factor of $\sqrt{2}$ (SHG) and $\sqrt{3}$ (THG). According to the polar characteristics of the LNT nanoparticles, the harmonic ratio resembles the volume nature of the harmonic generation. In other words, the results cannot be explained by surface SHG effects. According to the results of the optical band gap determination, it is possible to generate harmonics over a large spectral range. As the harmonic ratio validates the presence of a polar, i.e., bulk relevant effect, and further points to a negligible surface contribution of harmonic generation, we assume that the findings obtained here on the dispersion features can be transferred to bulk crystals without restriction. At the same time, it is reasonable to assume that the magnitude of the nonlinear optical response may be different according to a more complex intrinsic and extrinsic defect structure, including defect clusters. Compared with the edge compositions LN and LT, all results are found in accordance with the expectations. In this sense, it can be concluded that it is possible to use LNT in the same field of applications as already widely reported for LN and LT. Taking into account the recent finding for ferroelectric poling of LNT bulk crystals [47], this includes the preparation of periodically poled LNT structures for quasi-phase matching.

An added value of LNT, however, results for applications in nonlinear optics that benefit from the possibility of composition-dependent control of mechanical, electrical, and/or optical properties. As an example, we would like to mention here the zero birefringence crossing point that is dependent on composition and can, e.g., be shifted to appear at room temperature in LNT with x=0.94 [48]. Moreover, LNT may become an important material for integrated nonlinear optics of the next generation, nonlinear photonics, and quantum optics in the framework of the design of heterogeneous LNT-based structures. In this context, targeted engineering of parameters for matching the properties at the interface between two materials, e.g., LNT on insulator, is of particular importance. Here, LNT profits from the possibility of properly adjusting a variety of structural, electronic, and optical parameters, such as the lattice constant, the index of refraction, the band gap energy (as discovered in this contribution), stress, conductivity, etc., by means of composition control x, while keeping the nonlinear optical properties well-known from LN and LT, as demonstrated in this article. It can further be assumed that LNT has comparable mechanical and chemical properties to LN and/or LT and is, for example, non-hygroscopic and has a high mechanical hardness. At the same time, the already established wide spectrum of LNT synthesis protocols, including LNT waveguides [49], bulk crystals [16,50,51,52], LNT ceramics, LNT-glasses [53], LNT micro- and nanocrystallites [16,18], as well as the possibilities for nano- and microstructuring and doping, facilitate rather different fields of applications. However, we note that composition tuning may also have disadvantages. Apart from the band gap shift, which both limits the nonlinear optical window and favors multi-photon absorption processes, the precise adjustment of a desired composition could pose a particular challenge for crystal growth in terms of reproducibility.

## Figures and Tables

**Table 1:** Average crystalline sizes <$d_{\mathrm{XRD}}$> from XRD, weighted mean size <$d_{\mathrm{DLS}}$> from DLS, and the corresponding 5th and 95th percentile ranges of the DLS size distribution for the LNT nanoparticles with compositions x=0,0.25,0.5,0.75,1.

Composition *x*	<$d_{\mathrm{XRD}}$> (nm)	<$d_{\mathrm{DLS}}$> (nm)	[5th perc, 95th perc]
	Ref. [18]		(nm, nm)
0.00	206	277±8	[190, 396]
0.25	171	275±8	[220, 342]
0.50	97	299±8	[220, 459]
0.75	92	335±8	[220, 531]
1.00	80	402±8	[122, 825]

**Table 2:** Energies of the band gaps $E_{\mathrm{gap}}$ and corresponding wavelengths $\lambda_{\mathrm{gap}}$, $\lambda_{\mathrm{Fund},\min}^{\mathrm{SHG}}$, and $\lambda_{\mathrm{Fund},\min}^{\mathrm{THG}}$ (details in the discussion) for all LNT compositions x=0,0.25,0.5,0.75,1.0 determined from remission spectroscopy. Indirect allowed transitions are assumed and a linear fitting procedure with a Tauc plot is used.

Composition x	$E_{\mathrm{gap}}$ (eV)	$\lambda_{\mathrm{gap}}$ (nm)	$\lambda_{\mathrm{Fund},\min}^{\mathrm{SHG}}$ (nm)	$\lambda_{\mathrm{Fund},\min}^{\mathrm{THG}}$ (nm)
0	3.76 ± 0.02	330	660	990
0.25	4.10 ± 0.01	303	606	909
0.5	3.67 ± 0.01	338	676	1014
0.75	4.18 ± 0.03	297	594	891
1	4.53 ± 0.01	274	548	822

**Table 3:** Slopes $s_{\mathrm{SHG}}^{b}$ and $s_{\mathrm{THG}}^{b}$ as a result of fitting the function $I_{\left(\mathrm{SHG},\mathrm{THG}\right)}=c\cdot I_{\mathrm{Fund}}^{s_{\left(\mathrm{SHG},\mathrm{THG}\right)}^{a,b}}$ to the intensity dependencies of the second- and third-order harmonic signals for all LNT compositions under study.

Composition x	s${\;\;}_{\mathrm{SHG}}$	s${\;\;}_{\mathrm{THG}}$
0	1.83±0.01	2.84±0.03
0.25	1.81±0.02	2.97±0.05
0.5	2.01±0.03	2.84±0.04
0.75	1.98±0.02	2.82±0.05
1	1.91±0.02	2.70±0.07

## Data Availability

The data presented in this study are available on request from the corresponding author.

## Abbreviations

The following abbreviations are used in this manuscript:

LN	Lithium Niobate
LT	Lithium Tantalate
LNT	Lithium Niobate Tantalate
NLO	Nonlinear optical
OPA	Optical parametric amplifier
SHG	Second harmonic generation
THG	Third harmonic generation
FHG	Fourth harmonic generation
UV	Ultra-violet
VIS	Visual
NIR	Near-infrared
